# Western Australian Public Opinions of a Minimum Pricing Policy for Alcohol: Study Protocol

**DOI:** 10.2196/resprot.4815

**Published:** 2015-11-18

**Authors:** David A Keatley, Natacha Carragher, Tanya Chikritzhs, Mike Daube, Sarah J Hardcastle, Martin S Hagger

**Affiliations:** ^1^ School of Psychology University of Lincoln Lincoln United Kingdom; ^2^ National Drug and Alcohol Research Centre and the Office of Medical Education University of New South Wales Sydney Australia; ^3^ National Drug Research Institute Curtin University Perth Australia; ^4^ Public Health Advocacy Institute Curtin University Perth Australia; ^5^ Health Psychology and Behavioural Medicine Research Group School of Psychology and Speech Pathology Curtin University Perth Australia

**Keywords:** alcohol, addiction, policy, minimum pricing

## Abstract

**Background:**

Excessive alcohol consumption has significant adverse economic, social, and health outcomes. Recent estimates suggest that the annual economic costs of alcohol in Australia are up to AUD $36 billion. Policies influencing price have been demonstrated to be very effective in reducing alcohol consumption and alcohol-related harms. Interest in minimum pricing has gained traction in recent years. However, there has been little research investigating the level of support for the public interest case of minimum pricing in Australia.

**Objective:**

This article describes protocol for a study exploring Western Australian (WA) public knowledge, understanding, and reaction to a proposed minimum price policy per standard drink.

**Methods:**

The study will employ a qualitative methodological design. Participants will be recruited from a wide variety of backgrounds, including ethnic minorities, blue and white collar workers, unemployed, students, and elderly/retired populations to participate in focus groups. Focus group participants will be asked about their knowledge of, and initial reactions to, the proposed policy and encouraged to discuss how such a proposal may affect their own alcohol use and alcohol consumption at the population level. Participants will also be asked to discuss potential avenues for increasing acceptability of the policy. The focus groups will adopt a semi-structured, open-ended approach guided by a question schedule. The schedule will be based on feedback from pilot samples, previous research, and a steering group comprising experts in alcohol policy and pricing.

**Results:**

The study is expected to take approximately 14 months to complete.

**Conclusions:**

The findings will be of considerable interest and relevance to government officials, policy makers, researchers, advocacy groups, alcohol retail and licensed establishments and organizations, city and town planners, police, and other stakeholder organizations.

## Introduction

### Background

Excessive alcohol consumption has a direct negative impact on economic, social, and health outcomes. Regular moderate-to-heavy alcohol exposure is associated with numerous chronic health conditions, including liver cirrhosis, a range of cancers, and mental health problems [1-3]. Treating chronic harm from alcohol also places considerable burden on health care services [4]. In addition, acute patterns of alcohol consumption such as risky single-occasion alcohol consumption (ie, “binge” drinking) are associated with harmful outcomes such as drunk driving, violence, social disorder, and criminal behavior [5]. Binge drinking also has serious financial implications, including substantive costs for emergency services, such as ambulances or police services attending incidents caused directly or indirectly as a result of excessive alcohol consumption.

In Australia, studies by Collins and Lapsley [6] and the Foundation for Alcohol Research and Education have estimated the annual social and economic cost of alcohol consumption to be between AU $15 billion and AU $36 billion. Reducing the harmful outcomes associated with excess alcohol consumption is a frequently discussed component of Australian federal and state government policy agendas [4,7-10]. Policies influencing price are most effective in reducing population level consumption [11-14]. As a complementary policy to taxation, minimum pricing has received increased national and international attention in recent years [7,8,10,15,16]. Minimum pricing has attracted public health interest because it raises the cost of alcoholic beverages in proportion to their strength or alcohol content and, accordingly, targets beverages with high alcohol content sold at very low prices. While the policy has been implemented in a small number of countries to date, available empirical evidence indicates that population-level drinking is significantly reduced by minimum pricing [16]. However, evidence alone is not sufficient to ensure successful uptake of policy; public opinion is a key potential barrier to implementation [9].

Against this background, investigating attitudes to minimum pricing in Australia is an important research priority for public health advocates interested in policy avenues to reduce excessive consumption. An understanding of the Australian public’s attitudes and beliefs toward minimum pricing will provide critical insights into the likelihood of acceptability or opposition and inform public information campaigns that may pave the way for its introduction. The aim of this qualitative study is to investigate public beliefs and attitudes toward the introduction of a minimum price per standard drink policy. The study will be the first to investigate perceptions regarding alcohol minimum pricing in Australia and it will not only seek to provide evidence as to whether the public will support the introduction of minimum pricing, but also perceptions as to what circumstances or conditions may maximize its acceptability.

### Minimum Price Policies

A panoply of alcohol control policies have been proposed and implemented worldwide to reduce excessive alcohol consumption (see Babor et al for review [12]). Policies focusing on the price of alcohol have been found to be most effective in reducing excessive alcohol consumption [11,12,14]. Research has shown that population-level alcohol consumption is inversely related to the price of alcoholic beverages [8,17]. While alcohol duty and taxation have increased, so have average incomes such that alcohol, by comparison, has become more affordable. Alongside the increased affordability of alcohol, approximately 80% of the Australian population aged 14 and over report some level of alcohol consumption [4]. While annual rises in duty have reduced alcohol consumption, there is still considerable scope for consumers to access heavily-discounted alcohol. For example, consumers can substitute and alter their drinking habits to avoid higher taxes (eg, by switching to alternative, cheaper beverages such as cider [9]). Minimum pricing involves setting a “floor” or minimum price per standard drink, below which it would be illegal to sell alcohol. Unlike taxation, this policy cannot be circumvented by deep-discounting, below-cost strategies or promotions (ie, “buy-one-get-one-free,” “2-for-1,” or “multibuy” offers [18]). Modeling studies have indicated that minimum pricing would be effective in reducing excessive alcohol consumption and binge drinking [10,11,14,15,19-21]. Forms of minimum price policies have been implemented in a small number of countries such as Ukraine, Uzbekistan, Russia, the Republic of Moldova, provinces in Canada, and some US states (eg, Connecticut) [7,9,16,17,19].

While several leading public health organizations (eg, the National Alliance for Action on Alcohol, which represents 75 organizations) and organizations concerned with alcohol consumption in specific areas have voiced support for minimum pricing, public opinion on the policy is unclear. In fact, there is a dearth of studies worldwide investigating public opinion to minimum pricing [11,15,19,21]. Assessing public opinion and response to public health policies based on legislation, such as alcohol pricing policies, plays an important part in policy development. Government officials may be reluctant to implement policies that are unpopular or poorly understood by the public because of perceived fear that it might adversely impact them at the polls or that they might lose support of commercial interests [9].

Few studies have investigated public opinion toward minimum pricing [15]. An initial study in the United Kingdom revealed that responses of members of the general public to minimum pricing were “lukewarm” and “less than enthusiastic.” Participants indicated that they thought the policy would be ineffective and disliked. Participants also stated concern and skepticism regarding the aims and structure of minimum pricing, requesting greater transparency regarding where the additional revenue generated would be directed. Participants indicated that they would be more positively inclined toward the policy if the revenue generated was hypothecated to alcohol harm prevention and treatment strategies.

### Study Protocol

This protocol outlines the design of a study examining the knowledge, attitudes, and beliefs of members of the Western Australian (WA) public regarding a minimum price per standard alcoholic drink policy. The study will adopt a qualitative design to generate participant-led data on minimum pricing, including basic awareness, knowledge, and understanding of the policy and attitudes and beliefs toward its effect and possible introduction.

## Methods

### Design and Procedure

The study will employ a qualitative design, involving focus groups to gain in-depth and detailed insight into the awareness and knowledge of minimum pricing in members of the WA general public, their attitudes and beliefs toward the policy, and suggestions for increasing acceptability if the policy was introduced in WA. The study will be conducted over a 14-month period and recruit 10-15 focus groups comprising 8-10 adults in each group. Focus groups will last approximately 1 hour, led by a trained facilitator, and follow a semistructured standardized question schedule (Table 1) to ensure consistency and facilitate comparison and analyses.

The focus group schedule will be separated into 3 main parts. First, participants will be asked to indicate their understanding of the phrase “minimum pricing policy” with respect to alcohol. After assessing the participants’ knowledge, the facilitator will then provide a clear-language explanation of the policy for all participants. Information given by the facilitator will include a clear outline of the proposal, as well as previous evidence and findings related to the proposal. Second, the facilitator will subsequently enquire about participants’ attitudes and beliefs toward minimum pricing. Third, focus group participants will be asked to consider ways in which the policy could be made more acceptable and effective.

In-depth discussion will be stimulated by the facilitator throughout each part of the discussion. Data will be recorded on 2 voice-recording machines placed strategically to capture all voices in the room. The facilitator will encourage participation in an autonomy-supportive manner and prompt participants to be candid in their views and freely elaborate on their responses. Visual aids will also be introduced and explained to participants to assist with their understanding of how the minimum price policy will affect the price of alcoholic beverages.

### Participants

Eligible participants will include WA adults from a diverse cross section of backgrounds, including students, blue/white collar workers, minority groups, unemployed, and retired workers. Individuals younger than 18 years of age will be ineligible to participate as they are not old enough to purchase alcohol in WA. Further, participants who are considered harmful drinkers, according to a screening tool administered prior to the beginning of the focus group (eg, Fast Alcohol Screening Test), will be excluded. Any harmful drinkers identified during the course of the recruitment phase will be referred to alcohol awareness and counseling services.

### Participant Recruitment

Participants will be recruited through targeted advertisements and the research team’s existing collaborative links with the community, including schools, local employers, local clubs and organizations, and job seeker’s pages in local newspapers and in Perth job centers. Advertising materials (eg, posters and emails) will be developed to inform potential participants of the study aims and encourage them to contact the primary researcher to join a focus group. Posters advertising the study will be disseminated across the Perth metropolitan area, as well as emails sent to a wider catchment area in the neighboring suburbs of Perth. Email addresses will be sourced through word-of-mouth and Internet sites of groups and clubs.

One focus group will be exclusively female as research suggests women are likely to hold particular views and beliefs regarding alcohol drinking [22]. An additional 3-4 focus groups will target young professionals recruited from companies that employ white and blue collar male and female workers, groups that have reported high levels of alcohol consumption [23]. We plan to conduct approximately 4 focus groups among older adults from different ends of the socioeconomic spectrum. We will also plan to conduct focus groups in a sample of unemployed people. Finally, we aim to conduct 2 focus groups comprising people from the most populous ethnic minority groups in Perth, namely people from Chinese (eg, 2.9% of the Perth population) and South Asian (eg, Indian, Pakistani, Bangladeshi; 0.8% of the Perth population) backgrounds or have these groups represented in the sample. These focus groups will reflect a diversity of views, attitudes, and views.

**Table 1:** Interview questions for minimum price policy focus groups.

Focus group topic	Key questions	Follow-up questions
Reaction to minimum pricing	What are you your immediate thoughts about the minimum price policy?	What do think about the idea of minimum pricing?
		Do you think it is a good idea? Do you think it will work?
		Are you in favor of it?
	What concerns, if any, would you have about minimum pricing policy of alcohol?	
	What information/conditions would you like about the minimum pricing policy of alcohol before it was introduced?	
	What do you think are the possible outcomes of a minimum pricing policy?	
	Do you think that introducing minimum pricing policy will actually reduce how much people drink?	
	Who do you think will be most influenced by price increases?	
	How do you think price increases might influence your drinking?	Would minimum pricing policy change how much or what you drink?
	Do you think a minimum pricing policy will reduce alcohol-related harm, crime, social disorder?	
	Is alcohol different to other commodities? Would you continue to drink excessively regardless of any price increases?	
	Do you think minimum pricing policy will affect poor and rich people differently?	
	What impact do you think minimum pricing policy of alcohol will have on underage drinking? Heavy drinkers?	
Would you support the introduction of a minimum pricing policy of alcohol?	What are your reasons for supporting the policy? Or not?	
	What are the possible advantages of introducing the policy?	
	What possible negative effects do you think the policy may have?	
	Do you think the policy is aimed at particular subgroups of the Australian population? What are your reasons for this?	
	Do you think the policy fairly or unfairly focuses on certain subgroups in society?	
	Do you think the policy will work?	
What factors do you think would make a minimum pricing policy more tolerable or accepted by Australians?	What do you think would make the policy more effective?	
	Are there any additional steps (eg, information, public education) you think the government could take to help make this policy more acceptable?	

### Data Analytic Method and Sample Size

Given the paucity of research regarding public perceptions about minimum pricing, the adoption of a qualitative approach using focus groups is appropriate and fits well with the general aim to provide a comprehensive overview of people’s knowledge, attitudes, and beliefs of minimum pricing and to construct a model of the factors affecting the acceptability of the policy. Our qualitative approach will be data-driven following recommendations in the literature by qualitative research methodologists [24,25].

Although the approach is not guided by theory, it is not atheoretical. Instead, we will use a theory-building, inductive approach rather than a traditional theory-testing, deductive approach. Specifically, data in the form of transcripts of focus group discussions on minimum pricing will be subjected to inductive thematic content analysis [24] for relevant themes that will not only give important information about people’s knowledge of the policy but also provide detail on the relationship between key factors regarding minimum pricing and its acceptability and effect on drinking behavior. Our approach assumes no predetermined categories as it is important that themes emerge from the data during analysis with the focus on providing an in-depth understanding of the themes rather than “smoothed down” generalizations.

Based on Lonsdale et al [15], 10-15 focus groups will be recruited, giving an approximate sample size of between 80 and 150 participants. Once 10 focus groups have been completed, a preliminary data analysis using inductive thematic content analysis will be conducted. At this stage, if no new themes appear to be gained through further data collection, data saturation will be deemed to have occurred and the data collection stage of the study will end. Data analysis and judgment on data saturation will be verified by 2 experienced researchers in qualitative analyses.

### Measures

A research question protocol will be developed (Table 1) to ensure every focus group is asked the same questions pertinent to the research aims. This protocol will be developed based on the advice of a project steering group, comprising experts on alcohol policy, researchers on alcohol behavior, and stakeholders. It will then be pilot tested on several members (at least 10) of the general public to ensure that there are no ambiguities or errors and that the transition between topics allows for proper in-depth exploration of the key issues.

## Results

The study is expected to take approximately 14 months to complete. This includes 2-3 months for the development of materials (eg, question protocol, study posters). Approximately 1-2 months will be required for advertising and recruitment, followed by 4-5 months of data collection. It is then expected to take between 3 and 4 months to transcribe the data, analyze and agree on emergent themes, and write the results into a coherent framework.

## Discussion

### Expected Outcomes

This study protocol outlines study methodology to investigate attitudes and beliefs toward minimum pricing and identify avenues for increasing acceptability in members of the WA general public. This study will be the first of its kind in Australia and add to a nascent literature exploring public opinion about minimum pricing [15]. Given that there is increasing support for the introduction of minimum pricing around the world—particularly in Canada, the United Kingdom, and Ireland—this study is especially timely. Findings will likely be of considerable interest to researchers, policy makers, and stakeholders interested in managing and curbing excessive alcohol consumption. Further, the methodology could be used by other research and policy groups interested in examining public opinion to minimum pricing.

Although the planned analysis will be inductive and focus on emergent themes generated from the data, such a process does not occur in a “vacuum” independent of other literature and previous research. Based on previous research we can therefore form a candidate list of themes that may emerge from the data [23]. This will be used as a starting point for comparisons but will not be the sole focus of the analysis. The following key themes are expected to emerge in terms of beliefs regarding the minimum pricing alcohol policy: improving health, promoting law and order, saving public money, violation of personal freedoms, and indirect taxation. We also anticipate that knowledge and understanding of the policy, how it differs from other alcohol pricing policies, and its effects on differing levels and patterns of alcohol consumption in Australia may also differ from research conducted in countries with a history of publicity and debate over the introduction of minimum pricing, such as the United Kingdom [15]. The lack of knowledge and information regarding the policy may mean that understanding that it would have negligible effect on moderate drinkers’ expenditure on alcohol [26] and, hence, support for the policy, may be compromised in this sample. In addition, ways of making the policy more acceptable will involve providing information about health, economic, and social benefits of the policy, such as illustrating how the policy might save money by reducing the economic burden on health service, providing information about the risks of drinking and current problems, giving practical advice on how the pricing might affect “within-limit” alcohol drinkers, and framing the changes as socially responsible [15].

### Strengths and Limitations

The unique perspective and in-depth exploratory qualitative approach are major strengths of the current proposed study. It is the first study to examine the public interest case for minimum pricing in Australia and will provide valuable information on the perceptions of the general public that may assist in the development of legislation and messages toward the introduction of the policy. Furthermore, the methodological approach facilitates in-depth exploration of participants’ views and beliefs, and the use of a predetermined question protocol enables group comparisons.

There are some limitations with the current research. First, the researcher will not blind the study or background research. It may be the case that participants in each focus group ask the researcher for his or her views of minimum pricing. In this situation, the researcher will be briefed to remain neutral, will remind the group that the goal of the research is to canvas opinion and redirect the discussion to eliciting participants’ views and beliefs. Having an informed researcher is also an advantage in the context of the current study as confusion has been found to surround minimum pricing [15]. Second, due to logistical and financial considerations, this study will focus on a sample of adults from WA. Coupled with the small sample, this means that any extrapolation of findings may not generalize to the wider Australian public.

## Abbreviations

WA: Western Australia
